# The preparatory phase for ground larviciding implementation for chocerciasis control in the Meme River Basin in South West Cameroon: the COUNTDOWN Consortium alternative strategy implementation trial

**DOI:** 10.1186/s13071-022-05300-z

**Published:** 2022-06-21

**Authors:** Relindis Ekanya, Elisabeth Dibando Obie, Louise Hamill, Sophie Thorogood, Raphael Awah Abong, Abdel Jelil Njouendou, Andrew Amuam, Bertrand Lontum Ndzeshang, Desmond Akumtoh Nkimbeng, Jerome Fru Cho, Mathias Eyong Esum, Peter Enyong, Joseph D. Turner, Mark J. Taylor, Samuel Wanji

**Affiliations:** 1 COUNTDOWN, Department of Microbiology and Parasitology, Faculty of Science, University of Buea, Buea, Cameroon; 2 COUNTDOWN, Research Foundation for Tropical Diseases and Environment, Buea, Cameroon; 3 COUNTDOWN, Department of Biomedical Sciences, Faculty of Health Sciences, University of Buea, Buea, Cameroon; 4 COUNTDOWN, Department of Tropical Disease Biology, Liverpool School of Tropical Medicine, Pembroke Place, Liverpool, UK

**Keywords:** Breeding site, Susceptibility test, Ground larviciding, Temephos, *S. damnosum*, Onchocerciasis elimination

## Abstract

**Background:**

Onchocerciasis control using ivermectin alone has been achieved in some endemic savannah zones of Africa. In the forest regions, the co-endemicity with *Loa loa* has led to severe adverse events (SAEs) resulting in poor adherence of community members to ivermectin mass drug administration (MDA). This may jeopardize achieving the interruption of transmission of onchocerciasis. Therefore, to accelerate the elimination of onchocerciasis in *L. loa* co-endemic zones, alternative treatment strategies (ATS) including ground larviciding may be necessary. This study aimed at identifying *Simulium* breeding sites, cytospecies, transmission profile, susceptibility of *Simulium* larvae to insecticide (temephos) and identification of some non-target aquatic fauna prior to the implementation of the COUNTDOWN consortium ground larviciding alternative strategy in the Meme River Basin in South West Cameroon.

**Methods:**

A topographic map and entomological survey were used to determine breeding sites. Larvae and adults were identified using standard identification keys. Susceptibility tests were carried out on collected larvae by exposing them to decreasing concentrations of temephos and assessing survival rates while the cytospecies were identified using cytotaxonomy. Various entomological indicators were assessed from dissected flies. Fishing was used as proxy to traps to assess some aquatic fauna at different sites.

**Results:**

Twenty-two breeding sites were prospected in the Meme River Basin with eight productive for larvae. A concentration of 0.5–0.1 mg/l temephos induced 100% larval mortality. As the concentration of temephos decreased from 0.05 to 0.0025 mg/l, mortality of larvae also decreased from 98.7 to 12%. Nine cytospecies were observed in the Meme River Basin; 13,633 flies were collected and 4033 dissected. A total of 1455 flies were parous (36.1%), 224 flies were infected (5.5%), and 64 were infective (1.6%). Aquatic fauna observed included *Cyprinus* spp., *Clarias* spp., crabs, tadpoles, beetles and larvae of damsel fly.

**Conclusions:**

Onchocerciasis is being actively transmitted within the Meme River Basin. *Simulium* larvae are susceptible to temephos, and nine cytospecies are present. Non-target fauna observed included fishes, frogs, crabs and insects. Besides treatment with ivermectin, vector control through ground larviciding may be a complementary strategy to accelerate onchocerciasis elimination in the study area.

**Graphical Abstract:**

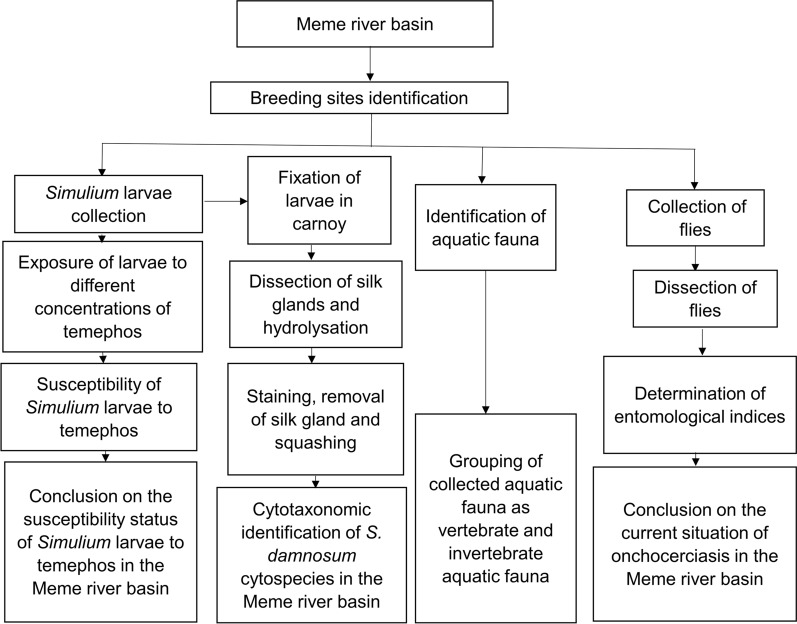

**Supplementary Information:**

The online version contains supplementary material available at 10.1186/s13071-022-05300-z.

## Background

Onchocerciasis, a parasitic disease caused by the filarial nematode *Onchocerca volvulus* [1], is a public health problem found in some of the world’s most disadvantaged communities in remote, rural sub-Saharan Africa. Adult worms live in subcutaneous nodules and produce millions of microfilariae during their reproductive life span (10–15 years). The microfilariae (mf) can live between 1 and 2 years in the skin, from where they are taken up by black fly vectors during a blood meal. Mf develops into infective larvae within black fly, which infect another person during a subsequent bite, thus maintaining the transmission cycle. Host inflammatory reactions to dead mf in the skin cause onchocercal dermatological disease and in the eyes ocular disease that may end in blindness. Because of the public health problem and socioeconomic effects of onchocerciasis, large-scale control programmes were created in the past with strategies for its control while in recent years strategies have been directed at its elimination [2]. In 11 West African countries, the Onchocerciasis Control Programme (OCP) aerial larviciding led to the control of onchocerciasis as a public health problem [3].

The advent of ivermectin and its donation by Merck & Co. (NJ, USA) [4] provided the opportunity for its large-scale use for the treatment of onchocerciasis by the African Programme for Onchocerciasis Control (APOC) in 20 endemic African countries. Ivermectin mass drug administration (MDA) has led to the interruption of transmission in some foci in Mali, Senegal, Sudan and Uganda [5–8]. Unfortunately, the same level of achievement has not been made in Cameroon, especially in the South West Region where onchocerciasis is co-endemic with loiasis.

In the South West Region of Cameroon, the persistence of onchocerciasis transmission is due to the existence of abundant breeding sites in fast-flowing rivers, which results in very high numbers of biting black flies transmitting the parasite and poor adherence of the community members to ivermectin mass drug administration [9, 10]. The poor adherence to ivermectin MDA is due to the fear of SAEs that follow ivermectin treatment of highly infected *Loa loa* individuals in co-endemic areas of onchocerciasis and *L. loa* [10, 11]. Faced with these challenges and coupled with the change in paradigm from onchocerciasis control to elimination, it was important for APOC to offer new approaches to improve or complement ivermectin MDA [12]. This led to adoption of alternative treatment strategies (ATS). These ATS include complementary vector control, enhanced community-directed treatment with ivermectin (CDTI), community-directed treatment (CDT) with drug combinations or new drugs and test-and-treat (TNT) strategies [13]. The ATS are essentially directed towards highly endemic onchocerciasis foci where the disease cannot be eliminated by annual CDTI alone.

The Meme River Basin of South West Cameroon has been under CDTI for > 18 years. A situational analysis of onchocerciasis conducted in this region by Wanji et al. [9] demonstrated persistent transmission despite ivermectin MDA with prevalence values as high as 52% [9]. This persistence in onchocerciasis transmission motivated the COUNTDOWN Consortium to evaluate implementation of one of the recommended ATS, ground larviciding, as a potential tool to accelerate the elimination of onchocerciasis in this endemic focus. Ground larviciding is known to significantly reduce the number of biting black flies, thus reducing disease transmission and infection rates. Pre-requisites to ground larviciding are: identification of breeding sites, susceptibility test of *Simulium* larvae to a larvicide of interest, identification of cytospecies in the river basin and identification of non-target aquatic fauna. In this study, we chose to use temephos (Abate^®^ 500 EC-BASF, Douala, Cameroon) as the larvicide. Temephos is an organophosphate larvicide which has been shown to be biodegradable and non-toxic to non-target fauna with a good carry during the rainy season. This article presents the breeding sites, identified *Simulium* cytospecies, current onchocerciasis transmission profile, susceptibility of *Simulium damnosum* larvae to temephos and a baseline survey of some non-target aquatic fauna present in the Meme River Basin prior to the implementation of the COUNTDOWN Consortium ground larviciding alternative strategy implementation trial.

## Methods

### Study area

Meme River Basin is situated in the rain forest of the South West Region of Cameroon approximately 60 km inland from the Atlantic Ocean. The topography is very diverse; the main feature is a mountain range (Rumpi Hills, characterized by a volcanic ridge culminating at 1764 m with a northeast orientation [14]. The volcanic ridge is broken by several valleys and constitutes a watershed that is the source for several rivers, the Manyu, Meme, Mungo and Ndian. These rivers go down steep slopes generating fast currents and appropriate flow rates that favour the establishment of permanent *Simulium* breeding sites conducive to high intensity of onchocerciasis transmission. The climate is characterized by 8 months of rainfall and a short dry season from December to March. The annual rainfall varies between 2500 and 4000 mm with annual temperatures ranging from 25 to 32 °C.

River Meme and its tributaries originate from the Rumpi Hills and flow down precipitous slopes some kilometres from the selected communities. The main tributaries of the Meme River are the Mbile River, which flows pass Marumba I and II, and the Uve River in Bakumba and Big Massaka. These tributaries create important *Simulium* breeding sites as they enter the Meme River.

### Identification of breeding sites

**Fig. 1:**
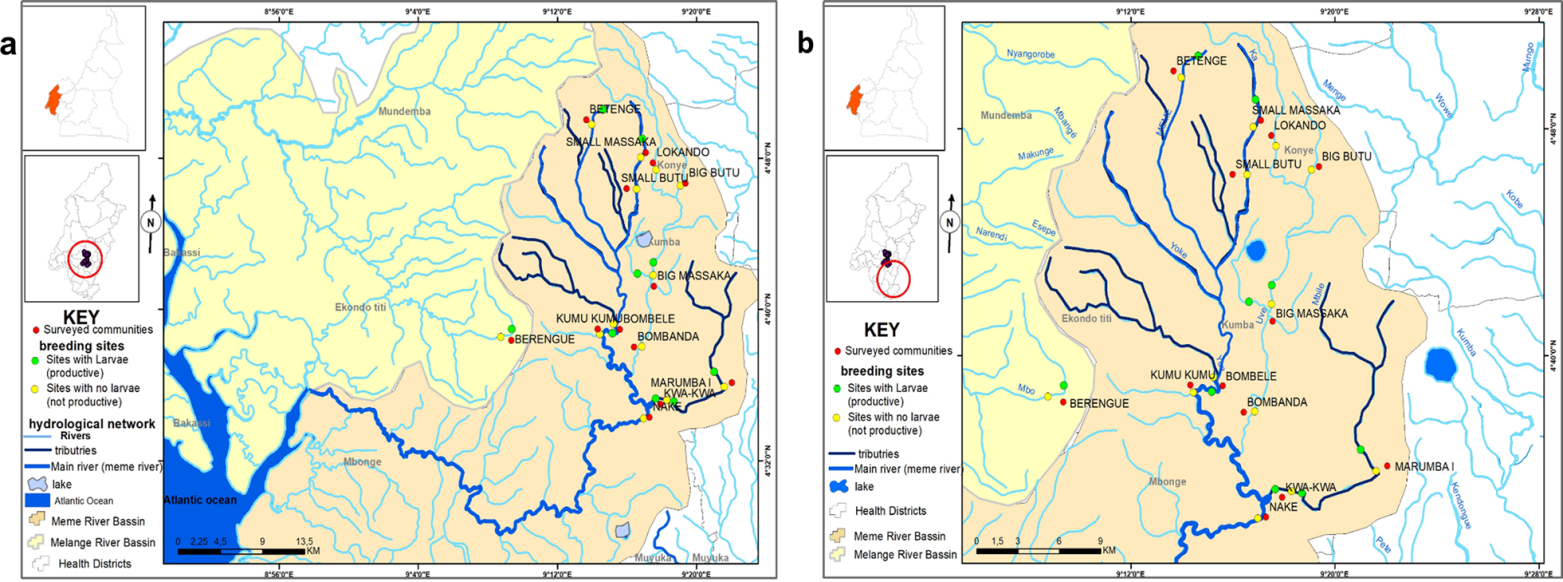
Map showing breeding sites geo-referenced using a GPS. **a** Map of the study and control area with prospected breeding sites. **b** Map showing productive and non-productive breeding sites of the study area

Potential *Simulium* breeding sites in the Meme River were selected using a topographical map of 1:200,000 scale [28] followed by an entomological survey to check for the presence of rapids, trailing vegetation and larvae to confirm that it is a breeding site. The Melange River in Ndian Division was set as the control area, which will be used to monitor the stability of entomological indicators after ground larviciding. The larvae and adults were identified using standard identification keys [15, 16]. All breeding sites were geo-referenced using a GPS. The co-ordinates were used to generate a topographical map of the breeding sites (Fig. 1a, b).

### Study sites

The study was conducted from March to July 2017. River Meme and its tributaries were surveyed, 20 breeding sites from 12 communities (Betenge, Small Massaka Lokando Big Butu, Small Butu, Big Massaka, Kumukumu, Bombele, Bobanda Kwakwa Nake and Marumba 1) were visited, but due to absence of larvae in some sites at the time of the study, only 8 sites from six communities (Betenge, Small Massaka, Big Massaka, Bombele, Kwakwa and Marumba 1) were chosen for fly and larvae collection. Due to accessibility, two sites near one community (Berenge) were surveyed in the Melange River Basin (control area). Flies were collected and dissected from one of the sites that had larvae to monitor the stability of entomological indicators in an untreated area after ground larviciding. Some sites in the Meme River Basin did not have enough larvae, so larvae for cytotaxonomy were collected only from three sites (Big Massaka, Bombele and Kwakwa). In the study arm, the breeding sites chosen were at:

- Marumba I (River Mbile, a tributary of Meme). Here, *S. damnosum* larvae were collected and a fly dissection point was set up at the river bank.
- Bombele, where the Meme River runs over several rock features developing rapids, which constitute breeding sites along its way to join the Atlantic Ocean.
- Kwakwa (along River Mbile, a tributary of Meme). Here, many larvae were found and a fly collection point was set up at the bank of the river.
- Big Massaka (River Uve, tributary of Meme). A survey of this tributary revealed many breeding sites which contained *S. damnosum* larvae. A catching point was therefore located in this community.
- Betenge, where the Meme runs. A fly collection and dissection point were located in this community
- Small Massaka (River Ka, tributary of Meme). A fly collection and dissection point were located in this community

### Susceptibility of *S. damnosum* larvae to temephos (Abate 500EC)

Larvae of *S. damnosum* were collected from several breeding sites, namely Marumba 1, Big Massaka, Bombele and KwaKwa in the Meme River Basin located in the South West Region of Cameroon. These larvae were exposed using a bowl containing local bottled water (Supermont) to ten concentrations of temephos (0.0005 mg/l to 0.5 mg/l) (Additional file 1: Text S1) for a period of 3 h [17]. After this, mortality was determined by observation of larvae motility response to stimulation [18].

### Dosage-mortality regression line

Adjusted mortality for each concentration was calculated utilising the control mortality frequency that ran in parallel with the test. If the control mortality was between 5 and 20%, then Abbot’s formula [19] was used to correct the mortality and ensure that other factors were not contributing to the mortality of the larvae. No adjustment was done if control mortality was < 5% and results were discarded for any testing with control mortality > 20%.

### Abbot’s formula

$${\text{Corrected test mortality }}\;{ = }\;\frac{{\left( {{\text{\% test mortality }} - {\text{ \% control mortality}}} \right){ }}}{{{ }\left( {{100} - {\text{\% control mortality}}} \right)}} \, \times {100}$$

Temephos dosage-dependent mortality curve was constructed using prism 7.0 (Graphpad, San Diego, CA, USA).

### Identification of cytospecies of *S. damnosum* in the Meme River Basin

Cytotaxonomy was used to identify the different cytospecies in the Meme River Basin. Cytotaxonomy requires the extraction and analysis of polythene chromosomes (giant chromosomes caused by cells that have undergone repeated rounds of replication) present in the silk gland of black fly larvae. Larvae for cytotaxonomy were collected from three breeding sites (Big Massaka, Bombele and Kwakwa) because the other breeding site did not have enough larvae for cytotaxonomy. The sixth and seventh stage larvae were collected and put in Carnoy’s fixative (3 parts ethanol: 1part acetic acid) that was freshly prepared at the riverside. A minimum of 30 larvae were put in 24 ml of Carnoy. The Carnoy’s solution was changed after 3 h and 24 h. The silk glands were dissected followed by hydrolysation to enable all the silk in the glands to be removed. Thereafter, chromosomes were stained for 5 min with Feulgen stain, which stains the DNA pink. Continual addition of orcien to the silk gland occurred to prevent dehydration. The gonads of the larvae were inspected to identify the sex. If male, the gonads are round, but if female, the gonads are elongated in shape. Squashing was done to separate the chromosomes while a light microscope was used to visualize and identify them. Photographs were taken at × 400. Identification of each larval chromosome was done using information from pictorial guide and identification key by Post et al. [20].

### Collection and identification of *Simulium* flies

*Simulium* flies were collected from seven different sites: Bombele and Betenge (River Meme), Big Massaka (River Uve), Kwakwa and Marumba I (River Mbile), Small Massaka (River Ka) and Berenge (River Melange). Residents of endemic communities for onchocerciasis were trained to carry out collections; *Simulium* flies landing on exposed legs for a blood meal were captured using suction tubes or mouth aspirators (locally adapted) before they bit [21].

Adult flies were either dissected to determine the parity and infectivity rate or stored in 80% alcohol for future use. *Onchocerca volvulus* transmission indicators (parous rate, infection rate, infectivity rate and monthly transmission potential) were computed after fly collection and dissection as described by Walsh et al. [22].

### Dissection of *Simulium* flies

Captured flies were killed using chloroform, counted and dissected in physiological saline under a dissecting microscope (Humanscope, HUMAN Diagnostics Worldwide, USA). Flies caught were dissected on an hourly basis to determine parity and infection status. This consisted of holding the fly with a needle in the thorax, piercing the abdomen with a dissecting needle at the posterior end and then pulling out the different internal organs to examine the fat bodies, state of the Malpighian tubules and ovaries to distinguish parous from nulliparous flies (nulliparous ovary is generally clear with follicles that are bigger and have voluminous fatty substance while parous flies generally have yellowish, spotted ovaries because of follicular relicts. The follicles are much smaller with little or no fatty substance). The head, thorax and abdomen of parous flies were further dissected separately and examined for *O. volvulus* developing larvae (L1, L2 and L3). Any larvae found were counted and recorded on a dissection sheet [23] and the entomological indices computed [22]

### Survey of some non-target aquatic fauna

Survey of some non-target aquatic fauna was conducted to obtain a picture mainly of the common fish species and also other common vertebrate as well as invertebrate non-target fauna to serve as indicators to guide measurement of any detrimental effect of temephos during ground larviciding. For this to be done, fishermen and -women were contacted to determine the species of aquatic vertebrates common in the Meme River and its tributaries. Thereafter, they were requested to carry out fishing exercises to determine the vertebrate aquatic fauna found at the different study sites. Locally made fishing nets were placed in water overnight to trap any available vertebrate aquatic fauna as modified from the protocol adopted by OCP in 1991 [24]. During the collection of *Simulium* larvae, invertebrate aquatic fauna present on the same substrate as the *Simulium* larvae were observed. Any aquatic fauna observed were grouped as either vertebrate or invertebrate. The vertebrate aquatic fauna were identified with reference to Temegne and Momo [25] while the invertebrate aquatic fauna were identified using a key by Danladi et al. [26].

### Data analysis

Fly collection data were entered in a template created in Epi info version 3.5.3 (https://www.cdc.gov/epiinfo/index.html), exported to Microsoft Excel and then later to SPSS version 24 for analysis. The different entomological indicators generated were biting densities, daily biting rate, monthly biting rate, parous rates infection rates, infective rates and monthly transmission potential. The chi-square test for trend was used to establish whether there was any association among parous rate, infection rate and infective rate.

Entomological indices were calculated using the following formulae:

- Parous rates were computed as the number of parous flies divided by the total number of flies dissected and multiplied by 100.
- Infection rates were computed as number of flies carrying any developmental form of *O. volvulus* larvae divided by the total number of parous flies dissected and multiplied by 100.
- Infective rates were computed as the total number of flies carrying infective larvae in the head divided by the total number of parous flies dissected and multiplied by 100.
- Biting density (number of flies collected at different hours plotted against the hours collected).
- Monthly transmission potential: (number of days in month × number of infective larvae)/number of fly collection days × (number of flies collected/number of flies dissected).

## Results

### Identification of breeding sites

Twenty-two breeding sites from 13 communities (Berenge, Betenge, Small massaka, Lokando, Big butu, Small butu, Big massaka, Kumukumu, Bombele, Bobanda, Kwakwa, Nake and Marumba 1) in both the control and study arms were prospected based on accessibility (Fig. 1a, b). Nine of these sites from seven communities (Berenge, Betenge, Small Massaka, Big Massaka, Bombele, Kwakwa and Marumba 1) had larvae (productive) at the time of prospection.

Breeding sites in Bombele and Betenge (River Meme), Big Massaka (River Uve), Kwakwa and Marumba I (River Mbile) and Small Massaka (River Ka) were identified as the main breeding sites of the study area; as such, fly and larvae collection points were set up at these sites. In the case of larval density, substrates were not calibrated but on average about ten larvae were found per substrate. *Simulium damnosum* was found to be the only anthropophagic species in the study area.

### Susceptibility test results

At a concentration between 0.5 and 0.1 mg/l, a mortality of 100% was recorded. It was observed that as the concentration of temephos decreased (from 0.05 to 0.00025 mg/l), mortality of larvae also decreased uniformly (from 12 to 98.7%) as seen in Table 1. Thus, *S. damnosum* larvae in these areas demonstrated good susceptibility to temephos (Abate 500EC).

**Table 1:** Susceptibility of *S. damnosum* larvae at various concentrations of temephos

Temephos concentration (mg/l)	Number of larvae tested	Average raw mortality% (SD)	Average adjusted mortality% (SD)
0.5	47	100 (0)	100 (0)
0.25	84	100 (0)	100 (0)
0.1	130	100 (0)	100 (0)
0.05	203	98.7 (1.9)	98.6 (2.1)
0.025	120	95.5 (4.2)	95.0 (4.7)
0.01	355	76.6 (14.0)	80.0 (15.1)
0.005	152	58.1 (34.2)	43.9 (40.5)
0.0025	252	44.2 (24.5)	39.7 (24.8)
0.001	187	30.7 (2.1)	23.4 (8.1)
0.00025	90	12 (7.1)	8.0 (1.4)
0 (Control)	577	9.8 (4.9)	–

**Fig. 2:**
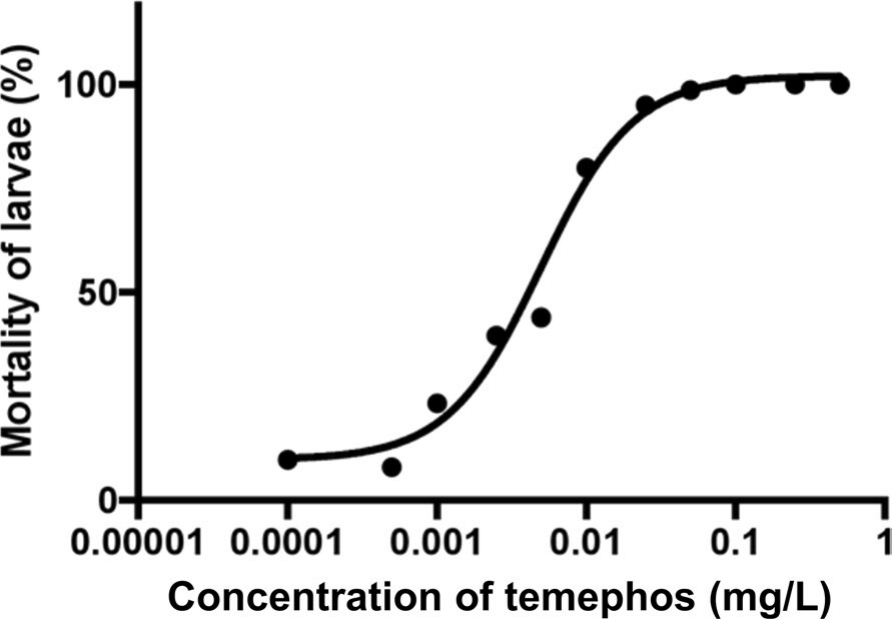
Dosage-mortality regression curve of concentrations. The control mortality was set to a concentration of 0.0001 mg/l as taking a logarithm of 0 is undefinable. The Hill slope (steepness of curve) was not constrained and included the control mortality

Dosage-mortality regression curve was plotted for susceptibility testing (Fig. 2). The control mortality was set to a concentration of 0.0001 mg/l as taking a logarithm of 0 is undefinable. This shows that the concentration of temephos required to kill 50% of larvae (EC50) is 0.005 mg/l and the concentration required to kill 95% of larvae (EC95) is 0.041 mg/l. The Hill slope (steepness of curve) was not constrained and included the control mortality.

### Identification of cytospecies in the Meme River Basin

**Fig. 3:**
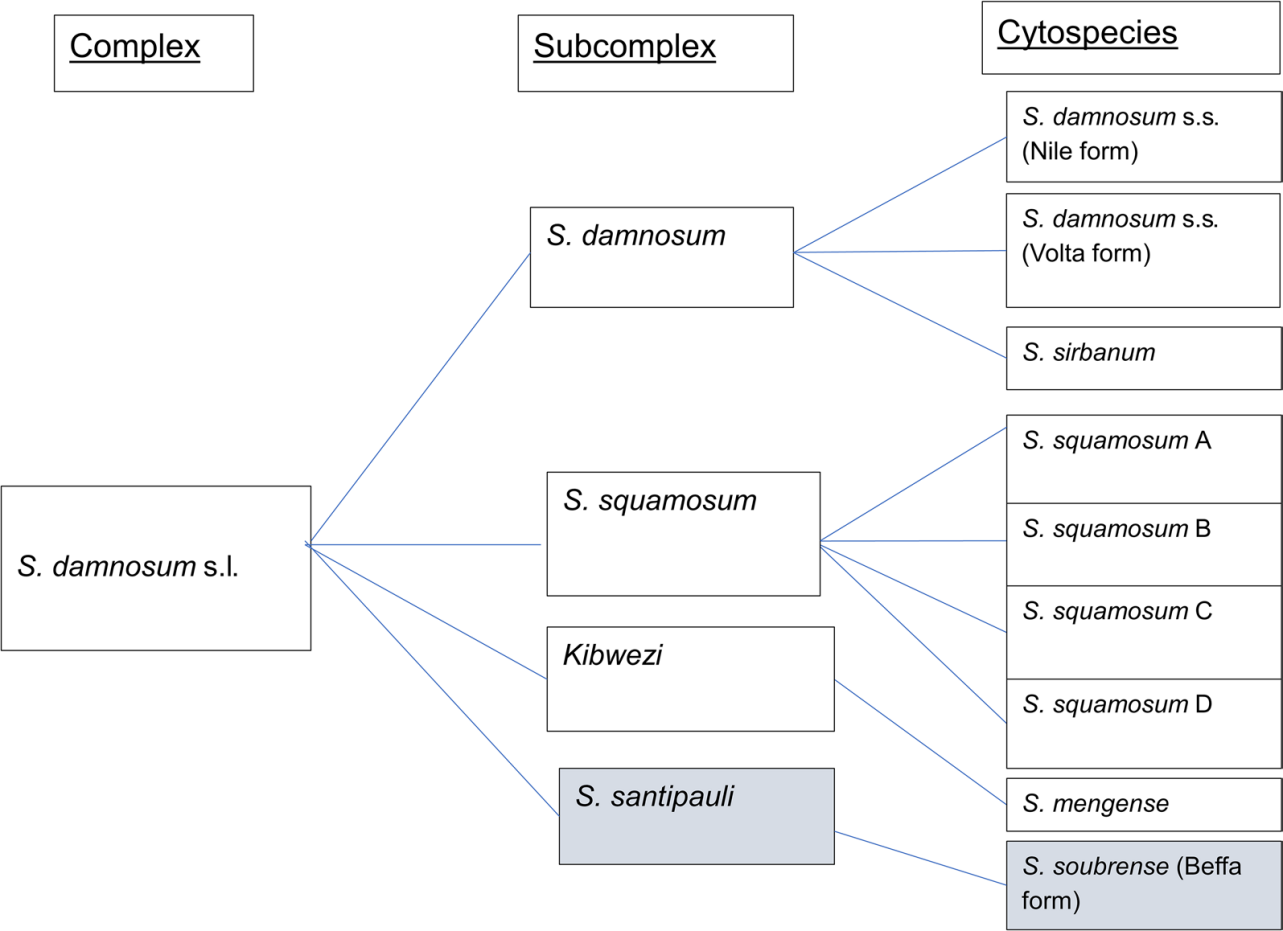
Observed cytospecies of *S. damnosum* in the Meme River Basin. The cytospecies *S. soubrense* (Beffa form) was observed but has not been previously described in Cameroon (in blue)

A total of 328 larvae were collected for cytotaxonomy with 40 from Bombele, 241 from Big Massaka and 47 from Kwakwa. Sixty larvae chosen for chromosome preparation due to their large size and white histoblast. This included 13 from Bombele, 29 from Big Massaka and 18 from Kwakwa. Chromosome preparations of 60 larvae were examined to identify high-quality images where the banding patterns on the chromosome were distinctive and clear. Additionally, some preparations were removed if the chromosomes were tightly coiled or broken. This led to the cytotaxonomic analysis of 15 larvae; 9 *S. squamosum* subcomplex larvae were found, which were three times the larvae found for any other subcomplex (Table 2). Four larvae from the *S. squamosum* subcomplex could not be identified as either male or female. The cytospecies *S. soubrense* (Beffa form) was observed but has not been previously described in Cameroon (Fig. 3, in blue).

**Table 2:** Classification of analyzed larvae into *S. damnosum* subcomplexes

		Breeding sites		
Subcomplex	Cytospecies	Big massaka	Bombele	Kwakwa
*S. damnosum* subcomplex	*S. damnosum* s.s. (Nile form)		Bom0517Tues3, F	
	*S. sirbanum*	BM0317Wed21, F		
*S. squamosum* subcomplex	*S. squamosum* (cytospecies undefined)	BM0317Wed18, U BM0317Wed22, F BM0317Wed23, U		KK0517LH2, U
	*S. squamosum* A		Bom0517Wed10, U	
	*S. squamosum* C	BM0317Thurs4, M BM0317Wed26, M		
	*S. squamosum* D	BM0317Wed25, M	Bom0517Wed10, M	
*Kibwezi*	*S. mengense*	BM0317Fri11, F		
*S. sanctipauli*	*S. soubrense* (Beffa form)	BM0317ST1, M		KK0517ST3, M KK0517ThuB, M

Abbreviations: M: male; F: female; U: unidentified

### Entomological indicators of *O. volvulus* transmission

Entomological indices of *O. volvulus* transmission in the study site are found in Table 3. A total of 13,633 flies were collected and 4033 were dissected. Of the flies dissected, 1455 were parous, giving a parous rate of 36.1%; 224 were infected (infection rate 5.5%) and 64 were infective giving an infective rate of 1.6%. Kwakwa had the highest parous rate (43.7%) while Marumba I recorded the lowest parous rate (17.8%). On the other hand, Bombele had the highest infection rate (10%) while Betenge had the lowest infection rate (1.4%).

**Table 3:** Entomological indices of *O. volvulus* transmission

Fly collection site	Total number of flies caught	Total dissected	No of parous flies (parous rate %)	No of infected flies (infection rate %)	No of infective flies (infective rate %)	L3/infective female	L3 /1000 parous females	Monthly transmission potential
Berenge	577	422	123 (29.1)	25(5.9)	10 (2.4)	3.8	308.9	211.9
Betenge	284	284	117 (41.0)	4 (1.4)	2 (0.7)	5.5	94.0	31
Big massaka	1292	216	54 (25)	9 (4.2)	6 (2.8)	2	222.2	1112.6
Bombele	4465	1025	425 (41.5)	103 (10)	22 (2.1)	4.8	247.1	742.7
Kwakwa	5241	1029	450 (43.7)	52 (5.1)	19 (1.8)	3.2	135.6	750
Marumba 1	540	353	63 (17.8)	10 (2.8)	3 (0.8)	3.7	127.0	268.6
Small massaka	1234	704	223(31.6)	21 (3.0)	2 (0.3)	2	17.9	27.2
Total	13,633	4033(29.6%)	1455 (36.1)	224(5.5)	64(1.6)			

### Biting density

**Fig. 4:**
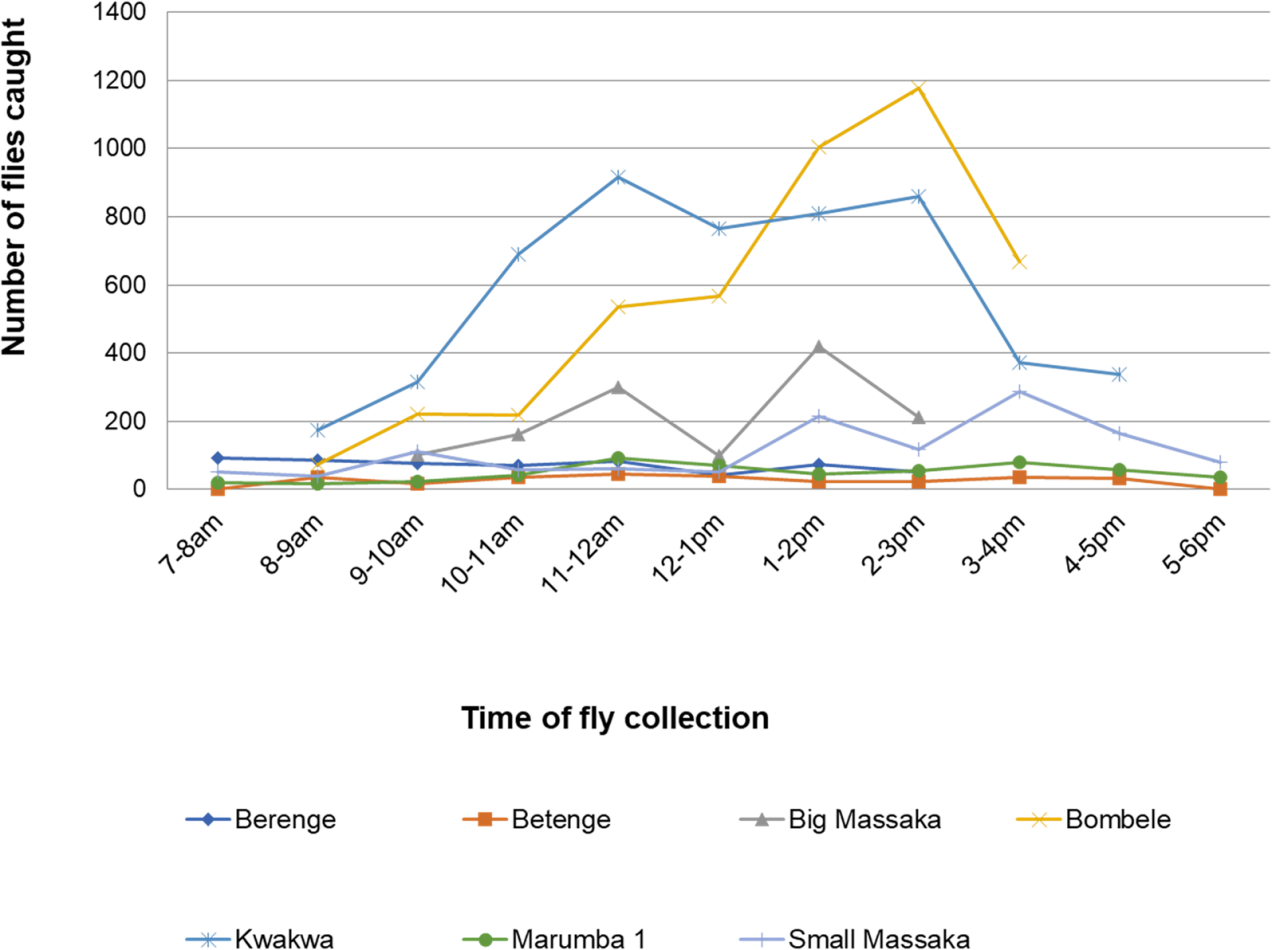
Biting density at different study sites. Peak biting hours for most of the communities were between 1–3 p.m. with as many as 1177 flies caught per hour at Bombele

More flies were caught during the afternoon hours with the peak biting hours for most of the communities between 1 and 3 p.m. This coincides with the peak farming hours. The most flies per hour were caught at Bombele with 1177 flies caught between 2 and 3 p.m. Biting density decrease towards the evening period (Fig. 4).

### Daily biting rate (DBR) and monthly biting rate (MBR)

The DBR and MBR for the various collection sites are shown in Table 4. The highest daily and monthly biting rates were recorded at Kwakwa (1311 and 40618 respectively) while the lowest daily and monthly biting rate was recorded at Betenge (142 and 4402 respectively).

**Table 4:** Daily biting rate (DBR) and monthly biting rate (MBR)

Fly collection site	Daily biting rate	Monthly biting rate
Berenge	289	8944
Betenge	142	4402
Big Massaka	1292	40052
Bombele	1117	34,604
kwakwa	1310	40,618
Marumba I	540	16,740
Small Massaka	309	9564

### Trend in entomological indicators within the study sites.

**Fig. 5:**
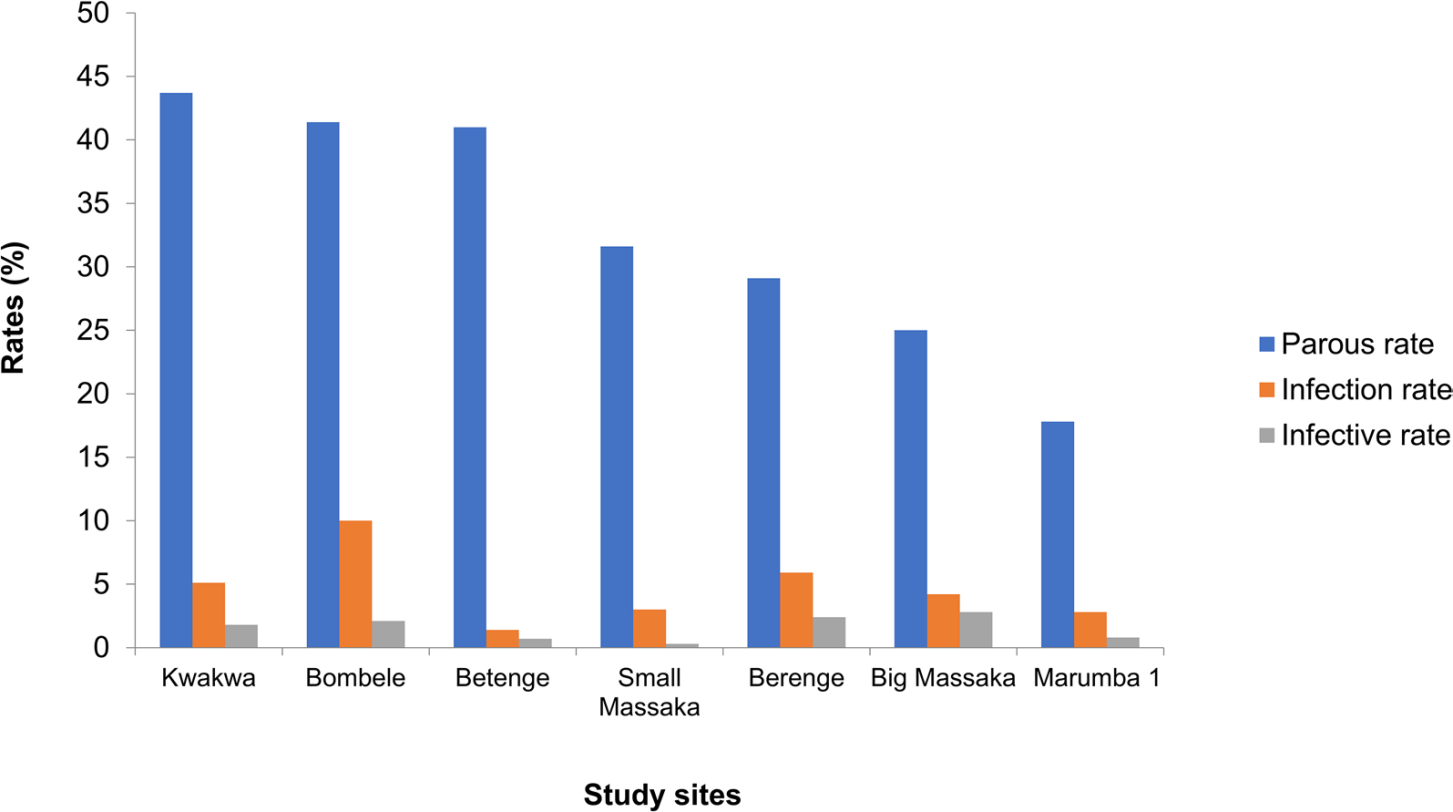
Variation in entomological indicators across study sites. A chi-square test of trend indicates that there was no statistically significant association (Chi-square test, *x*^2^ = 2.332, *df *= 1, *P* = 0.127) among parous rate, infection rate and infective rate

Figure 5 shows that there was some trend in parasitological indicators. Bombele with the highest parous rate also recorded the highest infection rates and eventually one of the highest infective rates. On the other hand, Betenge had a very high parous rate but recorded the lowest infection rate. As such, a chi-square test of trend indicated that there was no statistically significant association (Chi-square test, *x*^2^ = 2.332, *df *= 1, *P*-value = 0.127) among parous rate, infection rate and infective rate.

### Survey of some non-target aquatic fauna

The aquatic fauna found in the Meme River Basin were the same in all the study sites at the time of survey. This included two species of fish: *Cyprinus* (Mbanga fish) and *Clarias* sp. (African catfish). Other aquatic fauna observed were crabs and tadpoles. The presence of tadpoles indicated that frog species were also present though no frogs were captured. During the collection of *S. damnosum* larvae, aquatic Coleoptera (beetle) and the larvae of damsel flies (Odonata: Zygoptera) were also observed on the same substrate.

## Discussion

This study was designed to assess entomological indicators of onchocerciasis transmission and the susceptibility of *Simulium* larvae to temephos prior to the implementation of ground larviciding as an alternative treatment strategy for the acceleration of onchocerciasis elimination in the Meme River Basin. To realise this, we mapped the Meme River system, conducted a survey of the aquatic fauna, tested the susceptibility of *S. damnosum* larvae to temephos (Abate 500EC) and dissected a sample of adult black fly to determine entomological parameters.

In this study, exposure of black fly larvae to decreasing concentrations of larvicide (from 0.5 to 0.0005 mg/l) resulted in decreasing mortality of *S. damnosum* larvae. One hundred percent mortality was achieved at concentration exposures of between 0.5 and 0.1 mg/l and we determined EC_50_ of 0.005 mg/l. We therefore conclude that *Simulium* larvae are susceptible to ABATE 500 EC and this insecticide is effective for use in ground larviciding to accelerate the elimination of onchocerciasis in the study area. This is in line with the study carried out by Kalinga et al. [27] where there was 100% mortality of *Simulium* larvae to temephos between 0.129 and 0.34 mg/l pre- and 0.144–0.211 mg/l (95% CI, *P* < 0.05) post-treatment. The concentrations that were lethal to *S. damnosum* larvae in this study fall within the standard diagnostic dose of ≤ 0.4 mg/l for susceptible *S. damnosum* (s.l.) populations. This also shows the rapid activity of temephos, which has been shown to be highly efficacious against *S. damnosum* (s.l.) larvae at concentrations giving 0.05 mg/l at high river discharges (> 25 cubic m/s) and at 0.1 mg/l at low discharges (when insecticide carry is reduced). This is an indication that larvae from the Meme River Basin are highly susceptible to temephos.

Previously within Cameroon, there were eight cytospecies of *S. damnosum* recorded in the literature [20]. This record gives the most cytospecies that have been recorded in one country in West Africa [28]. This could be because Cameroon is situated in the Gulf of Guinea, which is known for its high biodiversity [29] with many different cytospecies occurring in proximity to different subcomplexes. In this study, the cytospecies *S. soubrese* (Beffa form) was found in two breeding sites (Kwakwa and Big Massaka). Notably, there have been no published studies that have recorded this cytospecies in Cameroon. However, this cytospecies has been recorded in Nigeria, which is a neighbouring country to Cameroon precisely in the Jos Plateau, which is 500 km from the Meme River Basin, and the Guarara River, which is 600 km away from the Meme River Basin [20]. The addition of *S. soubrense* (Beffa form) gives a record of nine cytospecies of *S. damnosum* found in Cameroon (Fig. 3).

There has been a decrease in infected flies from 12.9% in 2015 by Wanji et al. [9] to 5.5%. There has also been a decrease in infectivity from 3.9% in 2015 [9] to 1.6% obtained by this study. Results from this study show that onchocerciasis is still being actively transmitted in the Meme River Basin despite > 18 rounds of mass drug administration.

The availability of fast-flowing rivers in this basin contributes to the large numbers of black flies necessary for high transmission. In this pre-intervention baseline study, biting density was up to 36 bites/person/day, highlighting the need for a focal vector control [30].

In areas of high pre-intervention baseline infection endemicity, it has been suggested that vector control could be used as a complementary intervention strategy [31]. Modelling results using EPIONCHO and ONCHOSIM [32] suggest that in such settings vector control could be used in conjunction with ivermectin MDA to enhance effectiveness or to consolidate the gains made towards the path of onchocerciasis elimination. The aquatic fauna found in the Meme River Basin were similar in all the study sites. Both vertebrate aquatic fauna and invertebrate aquatic fauna were observed. It is expected that this aquatic fauna will not be affected since temephos is relatively non-toxic to fish and other aquatic vertebrates [33].

This study is limited in that it failed to collect quantitative pre-control data for non-target fauna, which would have been compared with post-quantitative data for the sake of environmental impact assessment.

## Conclusions

Onchocerciasis is being actively transmitted within the Meme River Basin. *Simulium damnosum* larvae in this river basin are susceptible to temephos and there are nine cytospecies of *S. damnosum* complex within the basin. Non-target fauna observed included fishes, frogs, crabs, and insects. Beside treatment with ivermectin, vector control through ground larviciding may complement and accelerate onchocerciasis elimination in the study area.

## Supplementary Information

**Additional file 1: Text S1.** Preparation of Abate dilutions.

## Data Availability

All data analysed during this study are included within the paper and supplementary information file.

## Abbreviations

APOC: African Programme for Onchocerciasis Control
ATS: Alternative treatment strategies
CDT: Community directed treatment
CDTI: Community directed treatment with ivermectin
COUNTDOWN: Calling time on neglected tropical diseases
DBR: Daily biting rate
DNA: Deoxyribonucleic acid
EC: Emulsifiable concentrate
MBR: Monthly biting rate
MDA: Mass drug administration
Mg/l: Milligram per litre
OCP: Onchocerciasis control programme
ONCHOSIM: Onchocerciasis transmission elimination simulation model
SAEs: Severe adverse effects
