# Supplementary material for: The preparatory phase for ground larviciding implementation for chocerciasis control in the Meme River Basin in South West Cameroon: the COUNTDOWN Consortium alternative strategy implementation trial
Source: Parasit Vectors. 2022 Jun 21;15:219. doi: 10.1186/s13071-022-05300-z (PMC9210632; doi:10.1186/s13071-022-05300-z)
Supplement: Supplementary file 1 — Additional file 1: Text S1. Preparation of Abate dilutions. [file 13071_2022_5300_MOESM1_ESM.docx]

**Preparation of Abate dilutions**:

Abate 500EC is provided at a concentration of 500g of active ingredient for every litre of product.

1g = 1000mg

Therefore, in 1L there is 500 000mg of temephos

10ml = 5000mg

10ml of abate in 990ml of water = 1L of 50mg/L. This is the stock solution

Before removing 10mls, the barrel was made to shake vigorously to ensure proper mixing of the solution.

The 50 mg/L working stock was used to prepare the following dilution series:

| Step | Concentration (mg/L, ppm) |
| --- | --- |
| 1. Stock solution | 50 |
| 1. 150ml of a, 1350ml water | 5 |
| 1. 500ml of b, 500ml of water | 2.5 |
| 1. 20ml of a, 980ml of water | 1 |
| 1. 150ml of b, 1350ml of water | 0.5 |
| 1. 150ml of c, 1350ml of water | 0.25 |
| 1. 150ml of d, 1350ml of water | 0.1 |
| 1. 150ml of e, 1350ml of water | 0.05 |
| 1. 150ml of f, 1350ml of water | 0.025 |
| 1. 150ml of g, 1350ml of water | 0.01 |
| 1. 150ml of h, 1350ml of water | 0.005 |
| 1. 150 ml of I, 1350ml of water | 0.0025 |
| 1. 150ml of j, 1350ml of water | 0.001 |
| 1. 150ml of h, 1350ml of water | 0.00025 |

The concentrations that were tested are highlighted above.
